# Knowledge of mental disorders in primary healthcare nurses in the Western Cape, South Africa

**DOI:** 10.4102/curationis.v48i1.2677

**Published:** 2025-05-16

**Authors:** John J. Musafiri, Million Bimerew, Jennifer Chipps

**Affiliations:** 1 School of Nursing, Faculty of Community and Health Sciences, University of the Western Cape, Cape Town, South Africa

**Keywords:** integration, knowledge, mental disorders, nurses, primary healthcare

## Abstract

**Background:**

Mental disorders remain a global health burden. The integration of mental health services into primary healthcare (PHC) can contribute to reduction of this burden. However, studies have reported PHC nurses’ lack of knowledge of mental disorders, which leads to their negative attitudes towards people with mental disorders preventing them from seeking help.

**Objectives:**

The objective of this study is to assess the knowledge levels of PHC nurses regarding mental disorders in the Western Cape, South Africa.

**Method:**

A quantitative descriptive survey was conducted. A self-administered questionnaire was used to collect data from a sample of 246 PHC nurses in the Cape Town metropole. Data were analysed using descriptive statistics, Chi-square tests and independent sample tests. A cut-off value of ≥ 80% was used to determine the sufficient levels of knowledge.

**Results:**

The average knowledge score (15.6; 78.0%) was below the cut-off value of ≥ 80%, indicating insufficient levels of knowledge. Sufficient levels of knowledge were found for over half of the respondents (139, 59.4%). Most of the respondents were knowledgeable about the symptoms of depression (221, 94.4 %), bipolar (217, 92.7%), schizophrenia (213, 91.0%) and anxiety disorders (209, 89.3%).

**Conclusion:**

Over half of the PHC nurses could identify the signs and symptoms of common mental disorders. However, the knowledge of dysthymia and psychosis requires more attention.

**Contribution:**

This study contributes to the existing body of knowledge in nursing practice and education related to mental disorders.

## Introduction

It is estimated that one in every four people worldwide will experience a mental disorder at some point in their lifetime (Tyerman et al., 2020; World Health Organization [WHO] 2024). Mental disorders, including schizophrenia, bipolar disorder, depression, and anxiety, are prevalent worldwide (Cao et al., 2024; Sahile et al., 2019) and continue to pose a significant public health challenge (WHO, 2022a, 2024). The WHO reported that 280m people experienced depression and 40m people were living with bipolar disorder worldwide, while approximately 24m people were affected by schizophrenia in 2019 (WHO, 2022b).

In the United States of America, around 46.6m adults suffer from mental disorders (Budenz et al., 2019), while these disorders account for 17% of the global burden of disease in China (Chang et al., 2021). The prevalence of these disorders has also been reported in Canada (Doran & Kinchin, 2019) and India (Maqbool et al., 2020). On the African continent, a study reported the prevalence of mental disorders in Nigeria, where 21.8m people may develop one of the mental disorders in their lifetime (Abdulmalik et al., 2019). This is alarming and requires an early detection of these disorders and adequate management. An increase in mental disorders has also been documented in Ethiopia (Ahmed et al., 2019) and Uganda (Mugisha et al., 2019).

Despite their high prevalence, mental disorders are often underdiagnosed or inadequately treated in many low-income countries (WHO, 2024). Within South Africa, common mental disorders, such as anxiety disorders, mood disorders such as bipolar and depression, schizophrenia and substance use disorders contribute significantly to the burden of disease (Anic & Robertson, 2020; Meyer, Matlala & Chigome, 2019; Mkhize et al., 2024; Sorsdahl et al., 2023). Although South African culture emphasises Ubuntu, meaning ‘humaneness’, people with mental disorders are often excluded from this ideal culture due to stigma and discrimination (Daniels & Isaacs, 2023). Consequently, people with mental disorders remain some of the most vulnerable members of society, often facing social isolation.

Mental disorders have far-reaching impacts on the lives of affected individuals, often impairing their academic performance as well as their social and occupational functioning (WHO, 2021). In response to the global burden of mental disorders, the WHO has recommended the integration of mental health services into primary healthcare (PHC) in high-income (Peritogiannis et al., 2014; Leung et al., 2018), middle-income (Meyer et al., 2019; Pandya et al., 2020) and low-income countries (Andersen et al., 2020; Kovess-Masfety et al., 2022). Given the great proportion of people presenting with signs and symptoms of mental disorders in PHC (Smith et al., 2024), screening for these disorders in PHC is crucial (Müller et al., 2024). The early detection of mental disorders among community members can enhance early recovery from these disorders and prevent functional impairment (Tay et al., 2018).

In PHC, the quality of mental health services depends on the knowledge of mental disorders among healthcare providers such as nurses and their beliefs and attitudes towards people with mental disorders (Kigozi-Male et al., 2023). A lack of knowledge about mental disorders can result in negative beliefs and attitudes among individuals towards people with mental disorders (Thornicroft et al., 2022). In this regard, a lack of knowledge about these disorders among healthcare providers such as nurses can hinder the good quality of mental healthcare.

Nurses are indeed considered as the frontline among healthcare providers, and they play a critical role in patient care, often being the first point of contact for patients and providing essential services across various healthcare settings (Sharma et al., 2021). Thus, PHC nurses are expected to have knowledge of mental disorders (Higgins et al., 2018; Kigozi-Male et al., 2023). However, a lack of confidence among non-mental health PHC nurses in caring for people with mental disorders poses a concern (Phungula et al., 2024). There have been reports of non-mental health PHC nurses having negative beliefs about people with mental disorders and negative attitudes towards them, which may constitute a barrier to the successful integration of mental health services into PHC (Seman et al., 2024; Yin et al., 2020). It has been postulated that these negative beliefs and attitudes might result from a lack of knowledge about people with mental disorders (Dalky et al., 2020; Latoo et al., 2021). This may impact accessing PHC services and prevent them from seeking professional help (Tay et al., 2018).

At the global level, studies have investigated nurses’ knowledge of mental disorders, primarily of nurses working in general hospitals, indicating a lack of knowledge among them, such as in Jamaica, China and the United States of America (Douglas et al., 2018; Hao et al., 2020; Kolb et al., 2023; Wang et al., 2023). Similarly, within South Africa, studies primarily focussed also on nurses working in general hospitals, finding a similar result of a lack of knowledge of mental disorders among these nurses (Mpetshu & Maritz, 2023). Some studies have investigated knowledge of mental disorders among PHC nurses, such as in Sweden, Vietnam, Jordan and Ethiopia (Bjorkman et al., 2018; Dalky et al., 2020; Murphy et al., 2018; Sahile et al., 2019), but only a few studies were found in South Africa (Kigozi-Male et al., 2023; Motaung, 2017). A study in the Free State province found that PHC nurses lacked mental health knowledge (Motaung, 2017), with a focus on PHC nurses’ perceptions of the prevention of mental disorders’ relapse following discharge. The study by Kigozi-Male et al. (2023) investigated PHC nurses’ mental health knowledge about mental healthcare seeking, the effectiveness of mental health treatment and recovery from mental disorders. This study did not focus on knowledge of the causes of mental disorders, their signs and symptoms. A study conducted in Limpopo found that PHC nurses caring for patients diagnosed with HIV lacked knowledge of screening the common disorders, such as depression and anxiety disorders, among these patients (Modula & Ramukumba, 2018). None of these studies conducted in South Africa focussed specifically on the importance of PHC nurses being able to recognise people presenting with mental disorders.

### Objective of the study

The objective was to assess the knowledge levels of PHC nurses regarding mental disorders in the Western Cape province of South Africa.

## Research methods and design

### Research design

A quantitative descriptive survey was used to collect quantifiable data related to PHC nurses’ knowledge of mental disorders (Creswell & Creswell, 2018).

### Research setting

This study was undertaken in 31 PHC facilities at which permission was granted to conduct the study. These facilities are a part of 47 PHC facilities located in the eight health subdistricts of the Cape Town metropole, one of the six districts of the Western Cape province of South Africa. However, eight of 31 PHC facilities are operated by City Health and 23 by the Provincial Department of Health. These facilities provide healthcare services to adult community members, and the baby clinics were excluded from this study.

### Population and sampling

The study population consisted of approximately 641 nurses permanently employed at 31 PHC facilities. A sample size of 246 nurses was calculated using Slovin’s formula (Yamane, 1967).

The sample was stratified across each subdistrict, and the percentage of the participants per each subdistrict was calculated, after which a simple random sampling was applied to determine the sample of nurses from each PHC.

### Instrument

A self-administered questionnaire included the ‘Mental Health Literacy Scale’ (MHLS) (with permission) (O’Connor & Casey, 2015). The scale was aimed at the general public and healthcare providers (Chao et al., 2020) and was modified for the PHC setting. The questionnaire consists of eight questions which are related to participants’ socio-demographic characteristics and 20 closed-ended statements on common mental disorders. Respondents were asked to rate a three-point Likert scale (‘disagree, uncertain, agree’) in terms of the accuracy of the statements. The majority of the 20 statements included signs and symptoms of different common mental disorders, and few consisted of definitions. The questionnaire was pre-tested with 10 PHC nurses who indicated that the terms included in this questionnaire were clear and readable. The Cronbach’s alpha was α = 0.80, which is reliable and acceptable (Taber, 2018).

### Data collection

Following ethics approval and receiving permission to use the health facilities for the study, the managers of the selected PHC facilities were contacted to request access to the nurses and arrange communication with them. The date and time for data collection were telephonically communicated to the participants 1 week before. The main data collection took place between December 2019 and January 2020. The researcher distributed the consent forms and self-report questionnaires to the participants who volunteered themselves to take part in the study. These questionnaires were completed in the presence of the researcher in a convenient venue at each PHC facility either during tea and lunch breaks or during times before and after official working hours. The completion of the questionnaire by each participant lasted approximately 20 min.

### Data analysis

Data were captured into the Statistical Package for the Social Sciences (SPSS) software version 27 (Polit & Beck, 2012). Descriptive statistics were used to describe and summarise the PHC nurses’ socio-demographic characteristics. Frequencies of the agreement were calculated and classified on the disorder statements as knowledgeable (the statement was correct), misinformed (the statement was incorrect) and lack of information when the statement was left unanswered. A total score out of 20 was calculated, and Bloom’s cut-off point of 80% was employed to classify sufficient knowledge of mental disorders (Benedict et al., 2022; Kamacooko et al., 2021). Respondents who obtained ≥ 80% were classified as having sufficient knowledge of mental disorders, and those who had a total score of less than 80% (< 80.0%) (Kamacooko et al., 2021) were classified as having insufficient knowledge of mental disorders. Chi-square tests were used to determine the association between demographic characteristics and PHC nurses’ knowledge scores of mental disorders (Polit & Beck, 2012). The independent samples Mann–Whitney U test was used to assess the relationships between PHC nurses’ prior exposure to people with mental disorders and knowledge. The independent samples Kruskal–Wallis test was used to ascertain whether there is a statistically significant difference between the medians of more than one independent sample, such as nursing categories’ knowledge of mental disorders.

### Ethical considerations

All ethical aspects were considered throughout the study (Gray & Grove, 2021) and were approved by the University of the Western Cape’s Biomedical Research Ethics Committee (reference number BM19/4/20). Permission to conduct the study at PHC facilities was obtained from the City Health Department and the Provincial Health Department. All the research participants’ rights, namely self-determination, privacy, anonymity and confidentiality, fair treatment, and protection from harm and discomfort (Gray & Grove, 2021), were explained to each of the PHC nurses who were invited to participate. The principle of respect for individuals and the principles of beneficence and justice, which are the three fundamental ethical principles, were applied (Gray & Grove, 2021).

## Results

### Socio-demographic characteristics

Out of the 246 nurses invited to participate, 234 completed the questionnaires, resulting in a 95.1% response rate. The average age was 49.79 (standard deviation [s.d.] 9.85) years, with the youngest being 22 and the oldest 65 years. Over a third of the respondents (81, 34.6%) were aged between 31 and 40, followed by respondents aged between 41 and 50 (69, 29.5%), respondents aged between 22 and 30 (40, 17.1%) and 51–60 (40, 17.1%). Most of the respondents were female (208, 88.9%). A total of 123 (52.6%) of the respondents were married or living together, with 111 (47.4%) being either single or divorced. Most respondents were registered nurses (140, 59.8%), followed by enrolled nursing assistants (50, 21.4%) and enrolled nurses (44, 18.8%). The respondents have worked in a PHC facility for an average of 7.7 years (s.d.7.1), with the shortest being less than a year and the longest being 37 years (median 5, 5 years). Nearly all of the respondents (199, 85.0%) indicated that they had provided care to a patient with a mental disorder at a PHC facility over the last year (Table 1).

**TABLE 1:** Socio-demographic characteristics (*N* = 234).

Demographic items	*n*	%
**Gender**		
Male	26	11.1
Female	208	88.9
**Marital status**		
Single	81	34.6
Married	112	47.9
Divorced	26	11.1
Widow	4	1.7
Partner	11	4.7
**Levels of education**		
Certificate	91	38.9
4-year diploma	57	24.4
4-year bachelor’s degree	71	30.3
Master’s degree	3	1.3
Other	12	5.1
**Nursing ranks**		
Enrolled nursing assistant	50	21.4
Enrolled nurse	44	18.8
Registered nurse	140	59.8

### Knowledge of mental disorders

**FIGURE 1:**
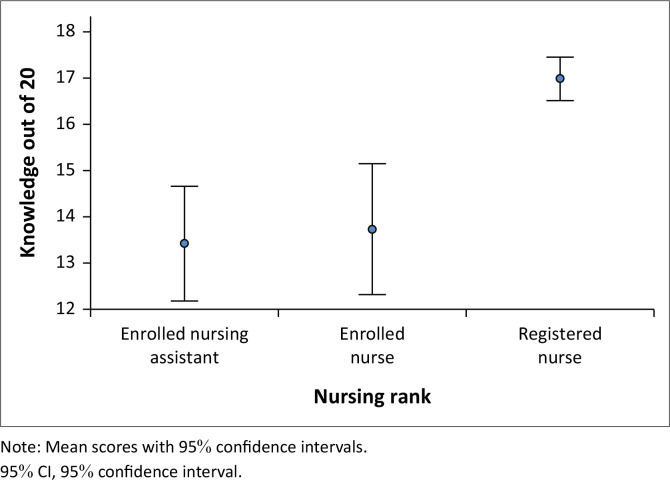
Mental health knowledge score and confidence interval.

The average score of knowledge of mental disorders was 15.6/20 (78.0%), which is below the cut-off value of ≥80%, indicating insufficient levels of knowledge. Nearly 60% (139, 59.4%) of 234 respondents scored ≥ 80%, meaning that they had sufficient levels of knowledge of mental disorders, but 95 (40.6%) had insufficient knowledge. Registered nurses had significantly higher scores than enrolled nurses and enrolled nurse assistants (17.0 vs 13.7 vs 13.4/20, respectively) (*K* = 42.0, *P* = 0.001) (Figure 1). Similarly, when comparing knowledge scores among three nursing categories, more registered nurses (104/140, 74.3%) scored ≥ 80% compared to enrolled nurses (18/44, 40.9%) and enrolled nursing assistants (17/50, 34.0%) (*X*^2^ = 32.5, *P* < 0.001).

Respondents with prior exposure to people with mental disorders had significantly higher knowledge scores (16.1 vs 13.0/20) for people with mental disorders (U = *p* < 0.001).

### Specific disorder knowledge

Most of the respondents were knowledgeable about depression (221, 94.4%), dementia (218, 93.2%), bipolar disorder (217, 92.7%) and schizophrenia (213, 91.0%). Similarly, 209 for anxiety disorder (89.3%), drug dependence (202, 86.3%), substance intoxication (199, 85.0%), phobia (190, 81.2%), personality disorder (186, 79.5%) and social anxiety disorder (185, 79.1%). The disorder with the highest proportion of respondents being misinformed was Alzheimer’s disease (25, 10.7%) and psychosis (32, 13.7%). The highest proportion of respondents lacking information was for cognitive behaviour therapy (80, 34.2%) and dysthymia (138, 59.0%) (Table 2).

**TABLE 2:** Levels of knowledge of mental health disorders (*N* = 234).

Knowledge items	Misinformed		Lack of knowledge		Knowledgeable	
	*n*	%	*n*	%	*n*	%
A person with depression experiences symptoms such as loss of energy, feelings of worthlessness, sleeping trouble, poor concentration, feelings of hopelessness and difficulty making decisions	3	1.3	10	4.3	221	94.4
A person with dementia suffers from a mental disorder in which progressive degeneration of the brain affects memory, thinking, behaviour and emotion	2	0.8	14	6.0	218	93.2
A person with bipolar disorder suffers from a mental disorder in which she or he experiences periods of elevated (i.e. high) and periods of depressed (i.e. low) mood	3	1.3	14	6.0	217	92.7
A person with depression suffers from a mental disorder in which she or he feels very sad and withdraws from society	11	4.7	9	3.8	214	91.5
A person with schizophrenia suffers from a mental disorder in which she or he experiences a different reality from that of the people around them	5	2.1	16	6.8	213	91.0
A person with an anxiety disorder has feelings of uncertainty, discomfort, worry about the future or tension that she or he experiences in response to an unknown object or situation	7	3.0	18	7.7	209	89.3
A person suffers from drug dependence, which is a substance use disorder in which she or he may experience withdrawal symptoms if the substance is withheld	4	1.7	28	12.0	202	86.3
A person with substance intoxication suffers from a substance-induced disorder in which she or he experiences psychological alterations of consciousness due to recent substance consumption	6	2.6	29	12.4	199	85.0
A person with a phobia experiences an excessive, unreasonable and persistent fear triggered by a specific object or situation	7	3.0	37	15.8	190	81.2
A person with a personality disorder suffers from a mental disorder, which makes her or him think, feel, behave or relate to others very differently from the average person	17	7.3	31	13.2	186	79.5
A person with a social anxiety disorder experiences an intense fear or anxiety about situations in which she or he may be under the scrutiny of others, she or he fears negative evaluation by others	8	3.4	41	17.5	185	79.1
Deliberate self-harm reported among persons with mental disorders, including depression, is perceived as the behaviour that a person uses to cope with difficult or painful feelings	12	5.1	46	19.7	176	75.2
A person with generalised anxiety disorder experiences and has difficulty controlling persistent and excessive anxiety and worry about a number of events or activities, such as work or social performance	16	6.8	43	18.4	175	74.8
A person with an eating disorder suffers from a mental disorder associated with severe disturbances in her or his eating behaviour	28	12.0	33	14.1	173	73.9
A person with delirium suffers from an acute, reversible, temporary disorder in which she or he may experience reduced awareness of or contact with the surroundings	14	6.0	49	20.9	171	73.1
A person with Alzheimer’s disease can remember an event that happened long ago, and the hallmark of this disease is the inability to form new memories	25	10.7	40	17.1	169	72.2
A person with psychosis’ mental capacity to recognise reality, remember, think, communicate with others, respond emotionally and behave appropriately is diminished	32	13.7	48	20.5	154	65.8
A person with agoraphobia experiences an abnormal fear of being in crowds, public places or open areas.	13	5.6	71	30.3	150	64.1
Cognitive behaviour therapy is designed to treat a wide range of mental disorders, including depression, and is a therapy based on challenging negative thoughts and increasing helpful behaviours	9	3.8	80	34.2	145	62.0
Dysthymia is a mental disorder known as persistent depressive disorder	12	5.1	138	59.0	84	35.9

## Discussion

The fact that PHC nurses are the first contact with people seeking professional help at the PHC level is crucial to knowing the signs and symptoms of common mental disorders (Modula & Ramukumba, 2018). The average score of knowledge of mental disorders was 15.6/20 which was below the cut-off value of sufficient levels of knowledge and nearly 60% of the respondents scored ≥ 80%, meaning that they had sufficient levels of knowledge of mental disorders. The study had a lower percentage of respondents (59.4%) who had sufficient levels of knowledge compared to 67.5% of intensive care unit (ICU) nurses in a study conducted in Australia (Wearea et al., 2019) and 91.0% of PHC nurses in India (Gandhi et al., 2019). Another study conducted in Ethiopia by Sahile et al. (2019) found that 368 (60.3%) of 610 PHC nurses had sufficient levels of knowledge of mental disorders. However, the knowledge cut-off point was set at 69.2%, which is lower than the 80% cut-off point in this study. In studies that did not use Bloom’s cut-off point but were similar in content, a study conducted on nurses in Ethiopia indicated that 50% of the respondents were knowledgeable of mental disorders (Mariam et al., 2016).

In the current study, sufficient levels of knowledge of mental disorders were more common in registered nurses due to their basic mental health training in their undergraduate nursing programme. Sufficient levels of knowledge of mental disorders were also noted among enrolled nurses and enrolled nurse assistants with prior exposure to people with mental disorders. Consistent with these findings, a study conducted in Australia found that nursing assistants had lower levels of knowledge of mental disorders than registered nurses (Gerace et al., 2018). An association between nursing categories and levels of knowledge of mental disorders was also documented in studies carried out in Malaysia (Eskandari et al., 2017), Taiwan (Chen et al., 2018) and the United States of America (Kolb et al., 2023). Regarding work experience, the findings of the current study are consistent with previous studies that reported nurses’ exposure to people with mental disorders contributing to their mental health knowledge in Taiwan and the United States of America (Chen et al., 2018; Kolb et al., 2023).

### Specific knowledge of disorders

In terms of specific disorders, most of the respondents correctly identified the definitions, signs and symptoms of mental disorders, which are common in South Africa such as depression, anxiety disorders, bipolar disorder, schizophrenia and substance use disorders. This is in contrast with a study in Ethiopia where only 30.7% of the respondents were unable to identify the signs and symptoms of common mental disorders (Sahile et al., 2019). The finding in our study is possibly related to the high number of respondents (85%) with prior exposure to people with mental disorders in PHC facilities and 60% of the respondents in the study being registered nurses. Registered nurses are expected to have mental health training in their undergraduate nursing programme in South Africa (South African Nursing Council, 2021), specifically for the nurses who qualified under the legacy programme with a specialist qualification in psychiatry.

Depression is one of the most common mental disorders, and 90% of our study respondents were knowledgeable about depression, but not about dysthymia (36%). Given the prevalence of depression across the world, PHC nurses should be able to screen depressive symptoms and manage the patients including referrals when needed (Mpetshu & Maritz, 2023). In contrast with our study, a lack of knowledge about depressive symptoms was documented among PHC nurses in Sweden (Wärdig et al., 2022), Vietnam (Murphy et al., 2018) and Kenya (Marangu et al., 2021). However, this study revealed that one-third of the respondents could not identify psychotic signs and symptoms, and nearly two-thirds lacked knowledge of dysthymia. Dysthymia, known as a persistent depressive disorder, is characterised by less severe symptoms than those with major depression (Abrams et al., 2017). Nurses should conduct dysthymia awareness among community members while focussing on the causes, risk factors, signs and symptoms (Gigy & Singh, 2018). However, there is a lack of studies that investigated PHC nurses’ knowledge of dysthymia. Similarly, studies that investigated PHC nurses’ knowledge of psychotic signs and symptoms are scarce.

### Limitations

Only knowledge of signs and symptoms of the common mental disorders in South Africa was examined in this study. The study did not look at PHC nurses’ knowledge about causes and risk factors and management of common mental disorders, apart from one question on cognitive behaviour therapy.

### Recommendations

Primary healthcare nurses are the first point of contact with people with mental disorders and they are responsible for providing integrated primary mental healthcare. This study recommends that PHC facility managers strengthen in-service mental health training for nurses to support the effective integration of mental health services into primary care. It is recommended that in-depth research be conducted to assess PHC nurses’ mental health knowledge including the causes and risk factors of common mental disorders and the management of these disorders.

## Conclusion

The findings of this study indicated that over half of the PHC nurses could identify the signs and symptoms of common mental disorders; however, important disorders such as dysthymia and psychosis require more attention.
